# Iatrogenic Severe Splenic Injury after Colonoscopy

**DOI:** 10.1155/2020/8824720

**Published:** 2020-10-06

**Authors:** Ikponmwosa Enofe, Jacob Burch, Julie Yam, Manoj Rai

**Affiliations:** ^1^Internal Medicine Resident, Michigan State University, Sparrow Hospital, East Lansing, MI, USA; ^2^Gastroenterology Fellow, Michigan State University, East Lansing, MI, USA

## Abstract

Colonoscopy is a low-risk procedure performed for screening and diagnostic purposes. About 15 million colonoscopies were carried out in the United States in 2012 with this number projected to increase. Injury to the spleen as a complication of colonoscopy is still a rather rare occurrence. We report a case of significant splenic injury, American Association of Surgery for Trauma (AAST) grade III with hemoperitoneum, in a patient following diagnostic colonoscopy, managed conservatively without the need for invasive or salvage surgical procedure.

## 1. Introduction

Colonoscopy is a low-risk procedure for screening, diagnostic, and therapeutic purposes [1]. Bleeding and colonic perforation are the most commonly described complications following colonoscopy [1–3]. Splenic injury is also a complication of colonoscopy, with only about 100 reported cases worldwide, making it a rare complication of colonoscopy [1, 2]. Here, we present a patient who developed significant splenic injury (grade III) [4] with hemoperitoneum as a complication of diagnostic colonoscopy. This case report highlights the importance of a high index of suspicion in patients with vague abdominal symptoms after colonoscopy and the need to increase awareness for splenic injury as a possible complication of colonoscopy, especially among family physicians, emergency room providers, and internists. We also provide an update in the management of this rare but potentially fatal complication of colonoscopy.

## 2. Case Report

A 72-year-old female presented to the emergency room (ER) with complaints of left upper quadrant (LUQ) abdominal pain and weakness 8 hours after a diagnostic colonoscopy was performed to investigate chronic constipation. Her past medical history was significant for two episodes of unprovoked deep vein thrombosis including one episode with associated pulmonary embolism for which she was on oral anticoagulation with warfarin. The patient was advised to stop warfarin 7 days before her colonoscopy and to use enoxaparin for bridging therapy. Prothrombin time (PT) and the international normalized ratio (INR) measured on the day of the procedure were 19.0 sec and 2.0, respectively. Colonoscopy was performed under monitored anesthesia care (MAC) with propofol. Normal findings were reported with no technical difficulties encountered nor any therapeutic intervention performed or biopsies taken. The duration of the procedure was 16 minutes. At discharge, she remained asymptomatic, and warfarin was resumed.

**Figure 1:**
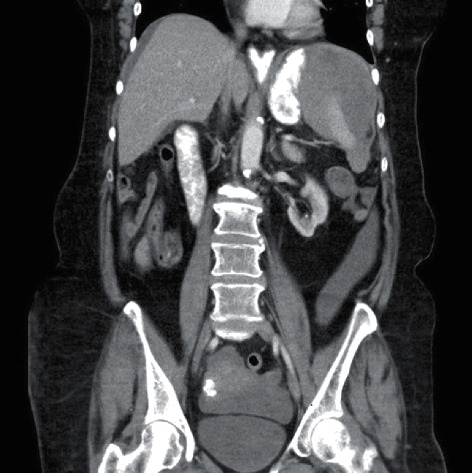
Coronal CT section of the abdomen and pelvis showing a large splenic hematoma.

**Figure 2:**
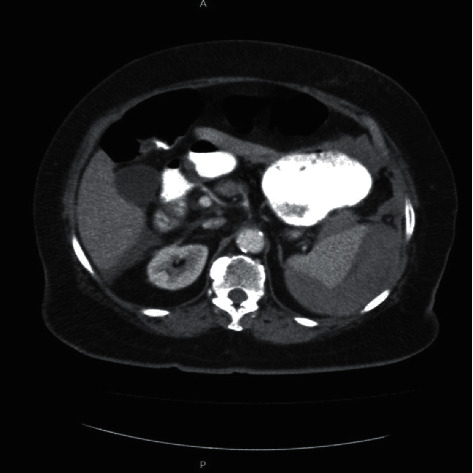
Axial CT scan section showing large subcapsular hematoma of the spleen with hemoperitoneum.

**Figure 3:**
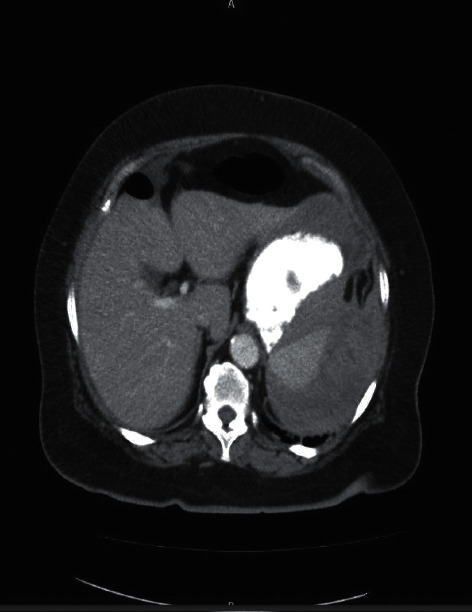
Axial CT scan, cross-section view, showing subcapsular hematoma of the spleen.

On return to the ER, examination revealed normal vital signs, a distended abdomen, and left upper quadrant (LUQ) abdominal tenderness. The rest of her physical examination was unremarkable. Laboratory examination showed a hemoglobin concentration of 8.1 g/dl, PT 15.3 s, INR 1.6, and activated partial prothrombin time (aPTT) of 22.3 s. Platelets were 322000 U/L at presentation. The rest of her labs were within normal limits. A computed tomography (CT) scan of the abdomen and pelvis with contrast revealed perisplenic heterogenous and hypodense fluid collection suspicious for hemorrhage or subcapsular hematoma with free fluid extending into the pelvis (Figures 1–3). No pneumoperitoneum was seen. Warfarin was discontinued, and she was managed nonoperatively with serial abdominal exams and hemoglobin measurements. Hemoglobin remained stable during hospitalization, and she did not require any blood transfusions. Warfarin was resumed on day 4. Hemoglobin was monitored for another 48 hours after resuming her on anticoagulation and remained stable at 9.0 g/dl.

The patient was discharged home with follow-up instructions to see her primary care physician for hemoglobin and INR check 48 hours following discharge. On follow-up 48 hours and a month after discharge, her hemoglobin was stable, and she had no abdominal discomfort or pain.

## 3. Discussion

Splenic injury is a rare complication of colonoscopy [1–3, 5–10]. The symptoms associated with postcolonoscopy splenic injury are nonspecific, and there is significant variability in the timing of onset of symptoms [1]; hence, it poses a diagnostic dilemma for clinicians [4]. Most often, it is diagnosed after it appears on the CT abdomen performed to rule out differentials including colonic perforation or retained gas following colonoscopy [5, 6, 11]. This may lead to a delay in diagnosis and an increased number of critical care admissions, with worse mortality outcomes [6, 12]. Most patients with splenic injury present within 24 hours after procedure [1–12]. However, some patients may present 10 to 14 days after procedure [2, 7, 13, 14] with the longest time reported from procedure to presentation being two weeks [2]. Left upper quadrant abdominal pain is the most commonly reported presenting symptom [6, 7]. Pain radiating to the left shoulders (Kehr's sign) may be present [6, 7] but has been reported to occur in as low as 34.4% of patients [1]. In some cases, patients may be completely asymptomatic [6, 7]. Clinical signs of hypotension, anemia, and elevated white blood cell count may be present [2, 4, 6].

The exact incidence of splenic injury following colonoscopy remains unknown [1, 6, 11] with a reported incidence of 1 per 6,000 to 1 per 100,000 colonoscopies performed [2, 6, 12]. This may be due to underreporting or misdiagnosis, and as such, its real incidence may be much higher [7]. Splenic injury following colonoscopy has a higher occurrence in women and older aged patients with a mean age of about 63 years [1, 7, 8, 11]. Risk factors associated with this complication are frequently a result of excessive manipulation, the presence of a redundant colon, history of previous abdominal surgeries, inflammation from previous intra-abdominal infections, and therapeutic colonoscopies. Splenomegaly, in addition to the use of propofol causing deep sedation, medications that increase bleeding risk (anticoagulants or antiplatelets), and inadequate bowel preparations, has been associated with a higher risk for injury to the spleen during colonoscopies [1, 2, 6–8, 13], although no direct causality has been identified with these risk factors and controversies still exist. In a review of 77 patients by Corcillo et al., anticoagulants and antiplatelets were not associated with an increased risk of injury to the spleen during colonoscopy. In the same review, splenomegaly and technically difficult colonoscopies, polypectomies, or biopsies were also not associated with a higher risk for injury to the spleen [8, 13]. Similarly, Singla et al. retrospectively reviewed over 102 cases of splenic injury occurring during colonoscopy reported in the literature over a 30-year period, and no association was observed between risk of splenic injury and the use of antiplatelet or anticoagulants, prior abdominal surgeries, and difficulty during colonoscopy [10].

The exact mechanism by which splenic injury occurs during colonoscopy is unknown. Maneuvers such as the endoscope slide by advancement, alpha maneuver, hooking of the splenic flexure, and straightening of the sigmoid loop may lead to avulsion of the splenocolic ligament resulting in laceration or rupture of the spleen [3, 4, 10–12, 15]. Furthermore, adhesions between the colon and the spleen from previous abdominal surgeries, inflammation, or infections have been suggested to increase traction from decreased mobility of the spleen and colon leading to splenic injury [4, 7, 8]. Precautionary measures such as placing patients in the left lateral position are recommended and are associated with a decreased occurrence in injuries to the spleen [1–6, 13]. This association is best explained by a reduction in the opposing tensions between the colon and the spleen from reduced traction in the splenocolic ligament and adjacent adhesions [1, 3, 7]. Other potential precautionary measures include avoiding looping [3], desufflation, and cautious sedation during the procedure [8]. Furthermore, few cases have reported splenic injury following upper gastrointestinal endoscopy (EGD) and endoscopic retrograde cholangiopancreatography (ERCP) [3, 12, 16, 17]. The exact mechanism of injury is unknown [15, 16] but was attributed to the traction of the greater curvature of the stomach and short gastric vessels [16].

Abdominal CT scan is the most accurate test used for diagnosing splenic injury [3, 5–11, 13, 14], with subscapular hematoma being the most common injury pattern encountered following colonoscopy [7, 13]. A CT scan has 98% specificity and sensitivity in diagnosing splenic injuries [11, 12, 14]. CT scans in hemodynamically stable patients help with grading, provide useful information in management, and exclude other possible differentials. Patients with splenic injury American Association of Surgery for Trauma (AAST) grade III or higher may need surgery [8]. Persistent hemodynamic instability remains the most critical determining factor for surgical versus nonsurgical management which may include radiologic embolization of the splenic artery [7, 11]. Unfortunately, up to 77.4% of patients presenting with splenic injury after colonoscopy may need surgical exploration with as high as 95% of patients needing splenectomy [8, 12]. More recently, the use of proximal splenic artery embolization (PSAE) in patients managed conservatively has been suggested to reduce the need for salvage surgical intervention [8] due to the high failure rates in nonoperative, conservative management compared to patients with injuries from blunt external trauma [4, 8]. There is however no sufficient data to suggest better outcomes and to advocate its use preemptively, as the standard of care in hemodynamically stable patients with grade III or less who are managed conservatively. Fortunately, our patient who had therapeutic INR at the time of colonoscopy and significant (grade III) splenic injury with hemoperitoneum was successfully managed conservatively without the need for invasive or salvage surgical procedure despite high failure rate of conservative management reported in the literature.

Despite several potential risk factors such as female sex, older age, use of propofol (causing deep sedation) during MAC, and use of anticoagulation in our patient, we cannot conclusively say why our patient had injury to the spleen during diagnostic colonoscopy, given there are no clearly identifiable risk factors that predict its occurrence. Due to weak evidence in data concerning risk factors and nonspecific symptoms [4, 13], we advocate more studies to better explain the contribution of each of these identified potential risk factors for developing injury to the spleen during colonoscopy.

## 4. Conclusion

Splenic injury still is a rare but sometimes fatal complication following colonoscopy. Given the lack of properly identifiable risk factors and variation in its presentation, this rare but potentially fatal complication can present a diagnostic dilemma for clinicians and requires a high index of suspicion in patients with postprocedural pain following colonoscopy. Greater awareness is needed amongst healthcare professionals, including family physicians, emergency room doctors, midlevel staff, and internists about this potentially deadly but rare complication following colonoscopy. Increased awareness may help improve outcomes and decrease mortality.
